# Idiopathic relapsing-remitting steroid-dependent pericarditis with transient constriction: a case report

**DOI:** 10.1093/ehjcr/ytaf041

**Published:** 2025-01-27

**Authors:** Fabio Cattaneo, Matteo Pasini, Giovanni Battista Pedrazzini

**Affiliations:** Cardiocentro Ticino Institute, Ente Ospedaliero Cantonale, Via Tesserete 48, 6900 Lugano, Switzerland; Cardiocentro Ticino Institute, Ente Ospedaliero Cantonale, Via Tesserete 48, 6900 Lugano, Switzerland; Cardiocentro Ticino Institute, Ente Ospedaliero Cantonale, Via Tesserete 48, 6900 Lugano, Switzerland; Department of Biomedical Sciences, University of Italian Switzerland, Via G. Buffi 13, 6900 Lugano, Switzerland

**Keywords:** Constrictive pericarditis, IL-1 receptor antagonist, Case report

## Abstract

**Background:**

Constrictive pericarditis is an uncommon complication of idiopathic pericarditis long thought to be irreversible, often requiring pericardiectomy to ensure recovery. Recently, transient constriction has been described in the setting of long-standing pericarditis with active inflammation.

**Case summary:**

We report the case of a 16-year-old male with an idiopathic relapsing-remitting pericarditis, refractory to non-steroidal anti-inflammatory drugs (NSAIDs), who developed constrictive physiology. Despite proper tapering, the patient developed five relapses during steroid treatment, which led to IL-1 receptor antagonist starting. After a long treatment with anakinra, constrictive physiology resolved. Disappointingly, discontinuation of interleukin-1 receptor (IL-1R) treatment was followed by a relapse of pericarditis, in the absence of signs of constriction.

**Discussion:**

Our case sustains that constrictive physiology may be a transient phenomenon, when the inflammation is still active and fibrotic transition has not been completed. When NSAIDs and steroid prove to be ineffective, IL-1R antagonist may represent a potential treatment for constrictive pericarditis, although there remains little evidence on de-escalation of IL-1R antagonist treatment in pericarditis.

**Learning points**

- Although usually thought to be irreversible, pericardial constriction can be a transient phenomenon, as long as the inflammation is still active and fibrotic transition has not been completed.
- Interleukin-1 receptor antagonist could represent a potential treatment for constrictive pericarditis, caused by persistent pericardial inflammation, avoiding unnecessary pericardiectomy.

## Introduction

Constrictive pericarditis is an uncommon complication of idiopathic pericarditis, which often requires complete pericardiectomy to ensure haemodynamic and myocardial recovery. Echocardiography, magnetic resonance imaging (MRI), and cardiac catheterization are usually employed in multi-modal evaluation for diagnosis.^1,2^ Echocardiographic signs consist of ventricular septal bounce, increased respiratory variation of mitral and tricuspid flows, and ‘annulus reversus’, whereas cardiac MRI is the gold standard to define pericardial thickening. In some cases, cardiac catheterization is necessary to achieve the diagnosis: ‘square root’ or ‘dip-and-plateau’ right ventricular pressure waveforms and end-diastolic pressure equalization are the diagnostic markers.^1–3^ Recently, some cases of pericarditis with transient constriction have been described, some of which resolved after treatment with IL-1 receptor antagonist.^3,4^

We report an infrequent case of an idiopathic relapsing-remitting, steroid-dependent pericarditis with transient constriction and prolonged but non-definitive response to anakinra.

## Summary figure

**Figure:**
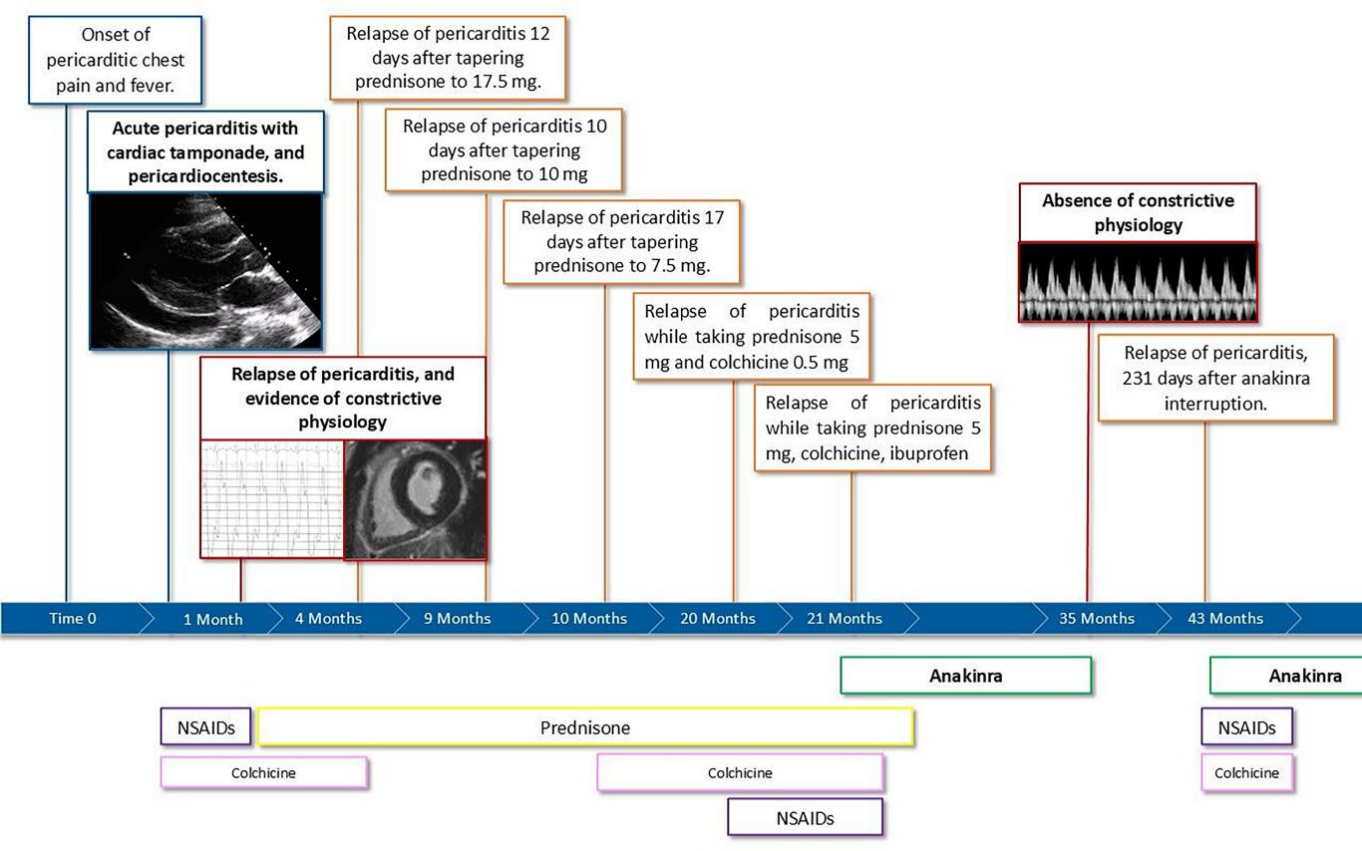


## Case presentation

A 16-year-old male presented to the emergency department for worsening of pericarditic chest pain and fever. The symptoms began 6 weeks earlier but were initially self-managed with non-steroidal anti-inflammatory drugs (NSAIDs) administration. No upper airways, gastrointestinal, genitourinary symptoms, or chest trauma were reported.

**Figure 1:**
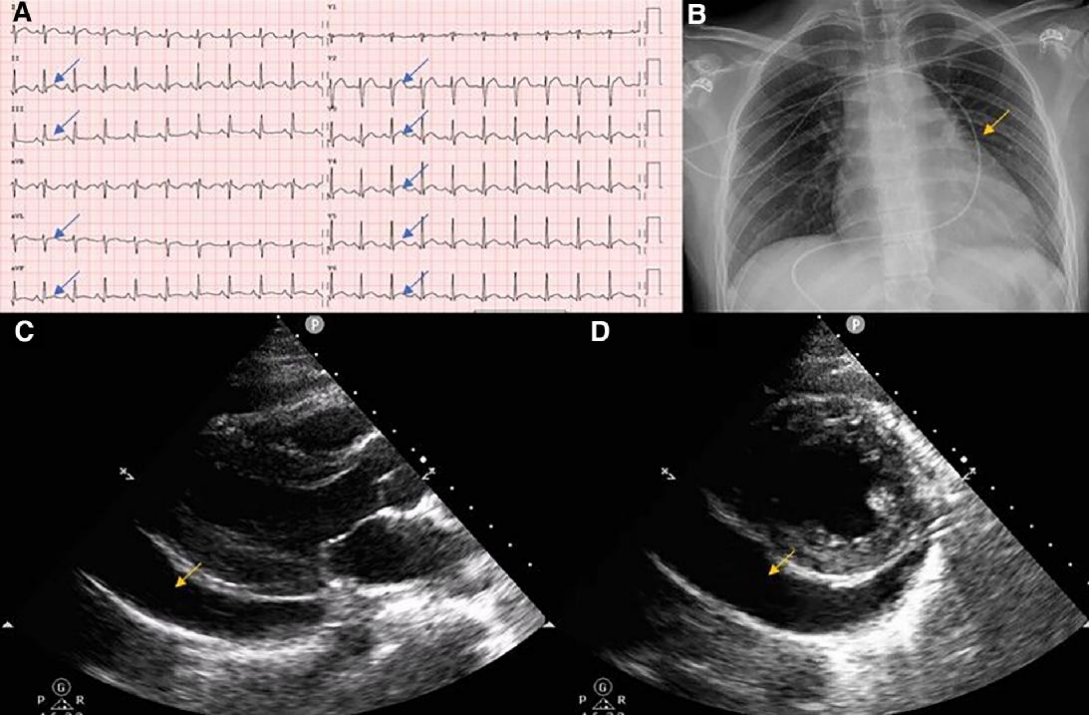
Electrocardiogram, echocardiography, and chest X-ray at diagnosis, showing widespread ST-elevation (arrows) (*A*) and haemodynamically relevant pericardial effusion (arrows) (*B*)–*D*).

At initial evaluation, tachycardia (122 b.p.m.) and tachypnoea were present. Blood pressure was 95/55 mmHg, and oxygen saturation was 94% on ambient air. Physical examination revealed muffled heart sounds, without adjunctive heart murmurs or pericardial rubs. Jugular venous distension was present, associated with mild ankle swelling. Pulmonary and abdominal examinations were unremarkable. Inflammatory response was seen at biochemical status: C-reactive protein was 150 mg/L [normal value (NV) <5], leucocytosis 20 000/µL (NV 4000–10 000/µL), with neutrophilia 17 160/µL (85%)]. N-terminal pro-B-type natriuretic (NT-proBNP) (114 ng/L, NV <125 ng/L) and Troponin T hs (9 ng/L, NV <14 ng/L) were normal, instead alanine aminotransferase (78 U/L, NV 10–35 U/L), aspartate aminotransferase (45 U/L, NV 10–35 U/L), and gamma-glutamyltransferase (153 U/L, NV 6–42 U/L) were elevated. Electrocardiogram (ECG) showed a typical pericarditic pattern, with widespread ST-segment elevation and PR tract depression, and bedside echocardiography reported a moderate pericardial effusion. Chest X-ray revealed cardiomegaly (*Figure 1*).

We made a diagnosis of acute effusive pericarditis and the patient required pericardiocentesis and intensive care unit admission for the presence of initial signs of cardiac tamponade: 500 mL of yellow citrine fluid was drained, and the pericardial fluid analysis demonstrated an inflammatory pattern. The aetiological evaluation excluded enteroviruses (coxsackieviruses and echoviruses), herpesviruses (Epstein–Barr virus, cytomegalovirus, and human herpesvirus 6), adenoviruses, parvovirus B19 or mycobacterial infection, and an autoimmune disease (anti-nuclear, extractable nuclear antigen panel, anti-neutrophil cytoplasmic, anti-cyclic citrullinated peptide antibodies, and rheumatoid factor were negative). Protein chain reaction for respiratory tract viruses on nasopharyngeal swab was negative.

**Figure 2:**
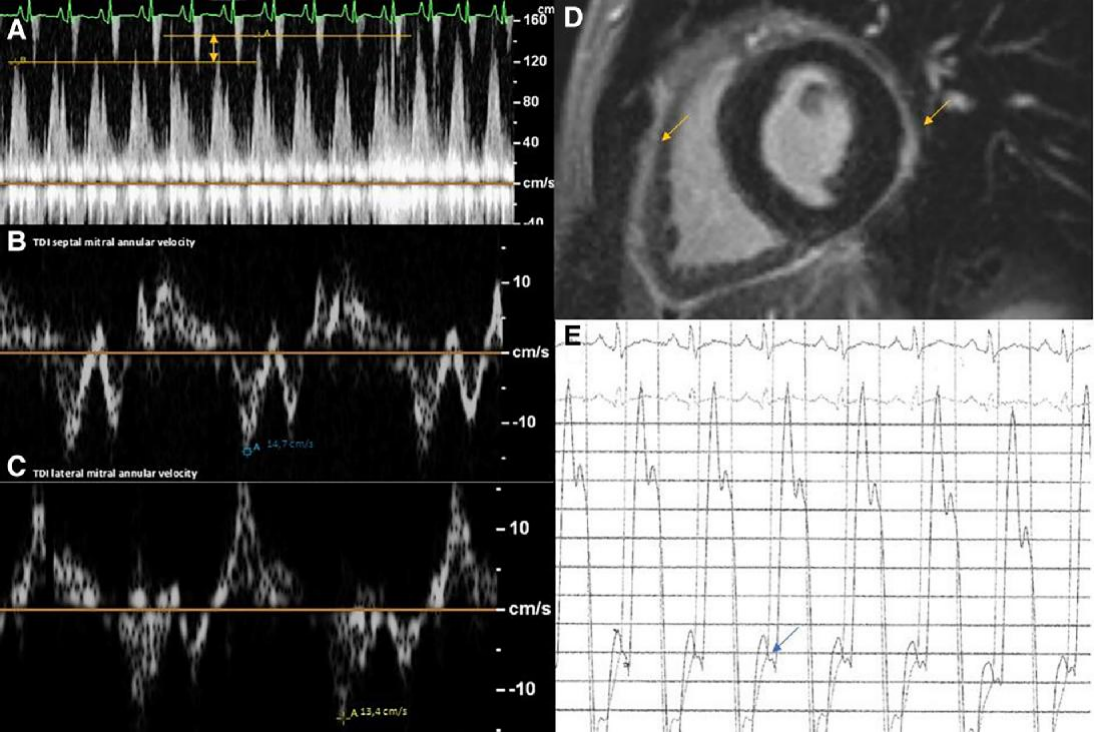
Echocardiography showing initial mitral flow respiratory variations (*A*) and normal mitral annular velocity with ‘annulus reversus’ (*B* and *C*). Cardiac magnetic resonance imaging (*D*) and cardiac catheterization (*E*) depicting increased pericardial thickness (orange arrows) and left ventricular and right ventricular diastolic pressure equalisation (arrows).

Initially, on a standard regimen of colchicine (0.5 mg bid) and NSAIDs (ibuprofen 600 mg tid), the patient experienced improvement of symptoms and inflammatory indices, and the pericardial effusion did not recur; therefore, he was discharged with a planned follow-up. Unfortunately, the patient experienced a relapse of pericarditic chest pain only 5 days after discharge with the evidence of worsening inflammatory indices, fever, and relapse of pericardial effusion (1 cm, circumferential) requiring readmission. Echocardiography raised suspicion of constrictive physiology, due to the evidence of septal bounce (see Supplementary material online, *Video S1*). There was a respiratory variability of the peak mitral E-wave velocity of 23%; furthermore on tissue Doppler imaging, the lateral E′ wave velocity (13.4 cm/s) was lower than the septal one (14.7 cm/s). Cardiac MRI (see Supplementary material online, *Videos S2* and *S3*) and cardiac catheterization confirmed some elements in favour of a constrictive pattern: pericardial thickening and similar tele-diastolic pressure profile in right and left ventricles (*Figure 2*). Specifically, during cardiac catheterization, it was observed that both ventricles overfilled and then stopped filling abruptly, resulting in the equalization of diastolic pressures in all chambers [mean diastolic left ventricle pressure 9 mmHg (NV <10 mmHg), mean diastolic right ventricle pressure 8 mmHg (NV 8–15 mmHg), mean right atrium pressure 8 mmHg (NV 0–6 mmHg), and pulmonary capillary wedge pressure 11 mmHg (NV 6–12 mmHg)]. Clinically, despite the lack of evidence of overt heart failure, NT-proBNP value (150 ng/L) was elevated. As suggested by current guidelines, we maintained colchicine treatment, and we switched from NSAIDs to oral steroids (prednisone 0.5 mg/kg), with a clinical and biochemical improvement leading to discharge.

**Figure 3:**
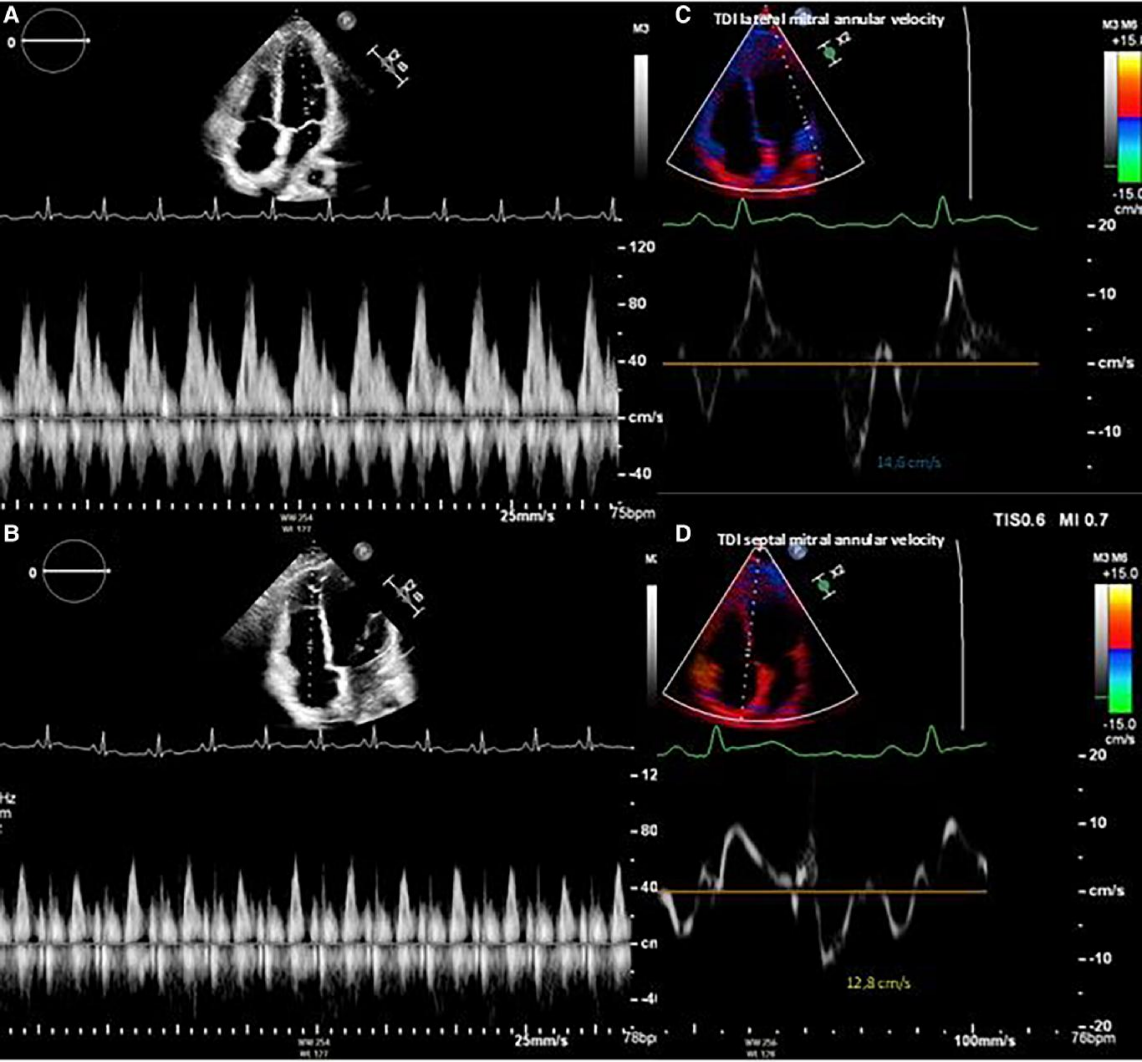
Echocardiography (*A–D*) showing the absence of constrictive pattern after IL-1R antagonist treatment.

The patient suffered from five more relapses of chest pain with biochemical correlate in the following 2 years, managed through steroid dose increase and careful tapering as with transient reintroduction of NSAIDs treatment. Each of the recurrences was characterized by the presence of pericardial chest pain, diffuse ST-segment elevation on ECG, and mild pericardial effusion. Autoimmune panel, viral serologies, and Quantiferon were repeated, with negative results. The specialist evaluation did not identify any rheumatological disease. In consideration of the steroid dependence with the impossibility to correctly taper and interrupt the treatment for relentless relapses, an interleukin-1 receptor (IL-1R) antagonist treatment was initiated (anakinra 100 mg/day), 21 months after the first hospital admission. Unfortunately, the introduction of an IL-1R antagonist was only possible after the appearance of side effects of steroid therapy, such as *striae rubrae*, since it is not approved by the national regulatory agency and health insurance has long refused to cover for the costs. This therapy offered a better control of the symptoms and biochemical inflammation, with complete remission of echocardiographic findings of constriction (*Figure 3*).

Despite slow tapering and prolonged therapy (14 months in total) with IL-1R antagonist, the patient experienced a new clinical and biochemical relapse leading to hospital admission, 8 months after anakinra interruption. The patient suffered pericarditic chest pain, along with diffuse ST-segment elevation and descending PR interval depression. A mild pericardial effusion (5 mm) and a C-reactive protein elevation of up to 105 mg/dL (NV < 5) were observed. A relapse of the constrictive pattern and pericardial effusion was excluded with transthoracic echocardiography. Anakinra was initiated with a new remission of the clinical and biochemical portrait. At the last follow-up, in the 10th month of anakinra therapy, the patient was free from consistent chest pain and inflammatory status at the blood testing.

## Discussion

Constrictive pericarditis is a rare disease, which develops in 1% of patients with idiopathic pericarditis, but can affect up to 20% of those with incessant or recurrent forms, as a consequence of sustained inflammation over time.^1,2^ Its treatment is challenging, and it often needs pericardiectomy to control the signs and symptoms of heart failure and improve the quality of life and prognosis of patients. Although long thought to be irreversible, transient pericardial constriction was first described in the 80s and after reported in several cases in which the constrictive physiology has resolved after adequate anti-inflammatory or immunomodulatory treatment.^3,4^

**Figure 4:**
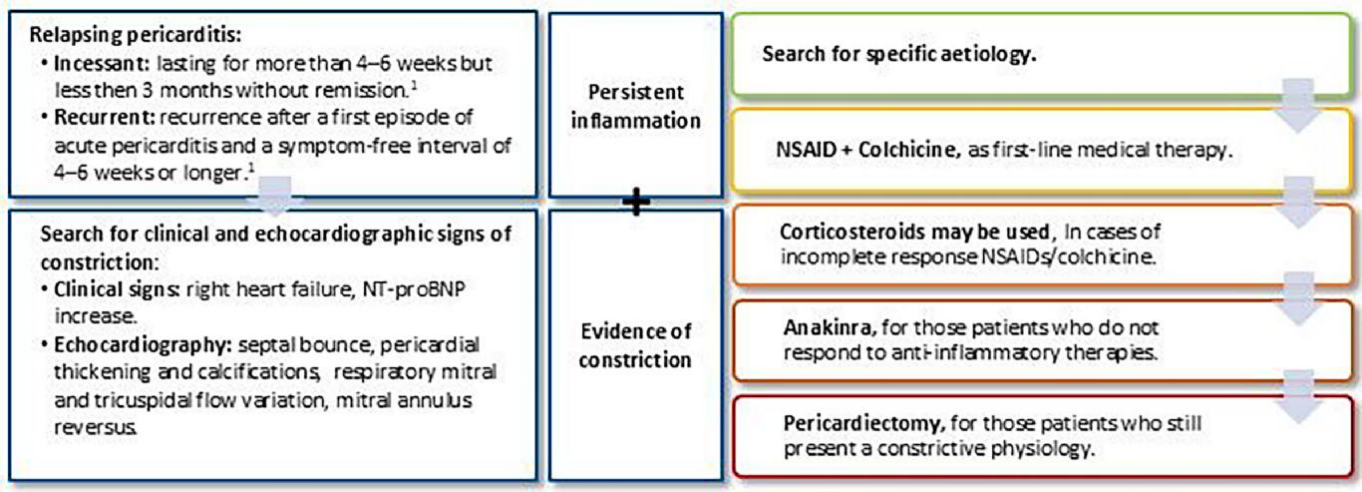
Diagnostic and therapeutic algorithm for relapsing-constrictive pericarditis management.

We report a case of steroid-dependent exudative-constrictive recurrent pericarditis, in which treatment with an IL-1R antagonist allowed control of pericardial inflammation leading to resolution of constrictive physiology, despite its introduction almost 2 years after diagnosis. Our case sustains the data that suggest that constrictive physiology may be a transient phenomenon, if adequately treated, when the inflammation is still active and fibrotic transition has not been completed. Since NSAIDs, colchicine, and steroids turned out to be unable to control symptoms and inflammation, anakinra was used to suppress chronic inflammation,^5,6^ surprisingly leading to complete resolution of the constrictive pattern. So, IL-1R antagonist may represent a potential treatment for constrictive pericarditis, caused by persistent pericardial inflammation, avoiding unnecessary pericardiectomy (*Figure 4*). It is possible that the beneficial outcome is mediated by the inhibition of NLRP3 inflammasome and IL-1, which have been demonstrated to be highly active in human pericarditis samples.^7^

Disappointingly, discontinuation of IL-1R treatment was followed by a relapse of pericarditis, in the absence of signs of constriction, which led to anakinra treatment reinstitution. Indeed, the weaning from anakinra remains an unresolved problem in the treatment of pericarditis, which still requires to be investigated and may be overcome in the future with the use of new IL-1R antagonists or systematic association with colchicine.^8–10^ Our patient still requires close follow-up to rule out future reappearance of a constrictive physiology.

## Supplementary Material

ytaf041_Supplementary_Data

## Data Availability

The data underlying this article will be shared on reasonable request to the corresponding author.

## Lead author biography

Dr Fabio Cattaneo graduated in 2014 from the Faculty of Medicine of the University of Milan and completed his residency in Cardiology in 2019 at the University of L'Aquila, Italy. He currently works as a junior consultant in the Cardiology Department of the Cardiocentro Ticino Institute in Lugano, Switzerland, dealing with general cardiology. His main areas of interest are heart failure, pericardial, and myocardial disease.

## Supplementary material

**Consent:** COPE’s best practice guidelines have been followed to obtain consent for publication, and the person being reported on is aware of the potential consequences of that reporting.

**Funding:** None declared.
